# Wound healing induced by new synthetic peptide, A7-1, in C57BL/6 mouse model

**DOI:** 10.1186/s12938-024-01247-7

**Published:** 2024-07-29

**Authors:** Gyu Sik Jung, Taehwan Park, JeongYeop Ryu, Joon Seok Lee, Jung Dug Yang, Ho Yun Chung, Kang Young Choi

**Affiliations:** Department of Plastic and Reconstructive Surgery, School of Medicine, Kyungpook National University, 130 Dongdeok-ro, Jung-gu, Daegu, 700-721 Republic of Korea

**Keywords:** Wound healing, Skin graft, C57BL/6 mouse model, Synthetic peptide, Experimental study

## Abstract

The effects of the novel synthetic peptide, A7-1, on wound healing and skin grafts were evaluated in a C57BL/6 mouse model. Two 15-mm wide circular skin excisions were made on the backs of mice and to each excision, 100 µM A7-1 or normal saline was applied daily. The treatments were applied and sutured for skin graft analysis. Digital photos were acquired on days 4, 7, 11, and 14 and fluorescein angiography was conducted. Wound sizes were verified using stereoscopic microscopy. Histological analysis was performed via hematoxylin and eosin staining and Masson’s trichrome staining. Western blotting was performed using vascular endothelial growth factor. Using a stereoscopic microscope, significantly faster wound healing (17.3%) and skin graft healing (16.5%) were observed in the A7-1 treatment group in comparison to that of the control. The angiogenesis was significantly faster in fluorescein angiography examination in wound healing (11%) and skin grafts (15%). However, the average completion of epithelization (overall time for wound healing), did not show any significant differences. In comparison to the control, the new protein, A7-1, led to significantly faster wound healing in the initial angiogenesis.

**Supplementary Information:**

The online version contains supplementary material available at 10.1186/s12938-024-01247-7.

## Background

Wound healing involves a series of complex processes, including interactions between various cells and tissues. The development of novel treatment modalities and a growing understanding of the pathophysiology of wound healing in the past three decades have led to remarkable advances, but wound healing and healing intractable wounds remain challenging [1–4]. In addition to the traditional method of using wet dressings, various methods and wound dressing materials, such as foam dressing, have been developed for healing skin-loss wounds caused by trauma or burns. Moreover, treatment methods using various materials including growth factors have also been developed and used in recent years. Materials and methods should be chosen after considering the various factors involved in wound healing, such as the duration of wound healing and pain reduction, as well as factors directly associated with the wound, such as its cause and condition [5, 6].

Various growth factors not only facilitate wound healing but are also being used for diagnostic and therapeutic purposes in various fields because of their diverse functions [7]. Among them, fibroin shows favorable adhesion to and growth in various cell types, including fibroblasts, keratinocytes, and osteoblasts, whereas when it is recombined with materials such as chitosan and poly-vinyl alcohol to form polymer complexes, fibroin facilitates wound healing by improving moisture and oxygen permeability [8, 9].

Therefore, the authors endeavored to identify functional motifs influencing cell adhesion among fibroin. Initially, 19 locations were selected from the entire 5263 amino acids of the fibroin protein heavy chain (P05790) of Bombyx mori, including the N-terminal, C-terminal, and repetitive sequences, for primary cell experiments.(Data not shown) The results revealed that the 18^th^ protein, GIPRRQLVVKFRALPCVNC, exhibited the most favorable activity. Subsequently, further investigation using various segments of the 18^th^ protein identified a 13-amino acid sequence, namely “RRQLVVKFRALPC,” as the most suitable. Consequently, A7-1 is synthesized by combining the C-terminal 13 amino acids of the fibroin protein heavy chain (P05790).

This study was conducted to investigate the effects of a novel synthetic protein A7-1 (A7-1 Sewon, Seoul, Republic of Korea) on wound healing. A7-1 was developed by modifying the fibroin heavy chain, which is composed of 13 amino acids (RRQLVVKFRALPC), and exists as collagen and glycosaminoglycan in the extracellular matrix. The minimal functional sequence was modified using homology and domain with silico analysis to determine the fibroin heavy chain, and the consensus sequence was then modified using pattern search for protein database with silico analysis to obtain A7-1.

Although A7-1 is known to facilitate tissue regeneration, vascular regeneration, wound healing, and engraftment, its distribution or expression in healthy human tissues remains unclear and no studies have examined its expression pattern in skin wounds. Accordingly, the novel protein A7-1 was administered to a C57BL/6 mouse wound healing–restricted contracture model and full-thickness skin graft model to determine its potential for improving wound healing [8].

## Results

### Measurement of wound healing rate

**Fig. 1:**
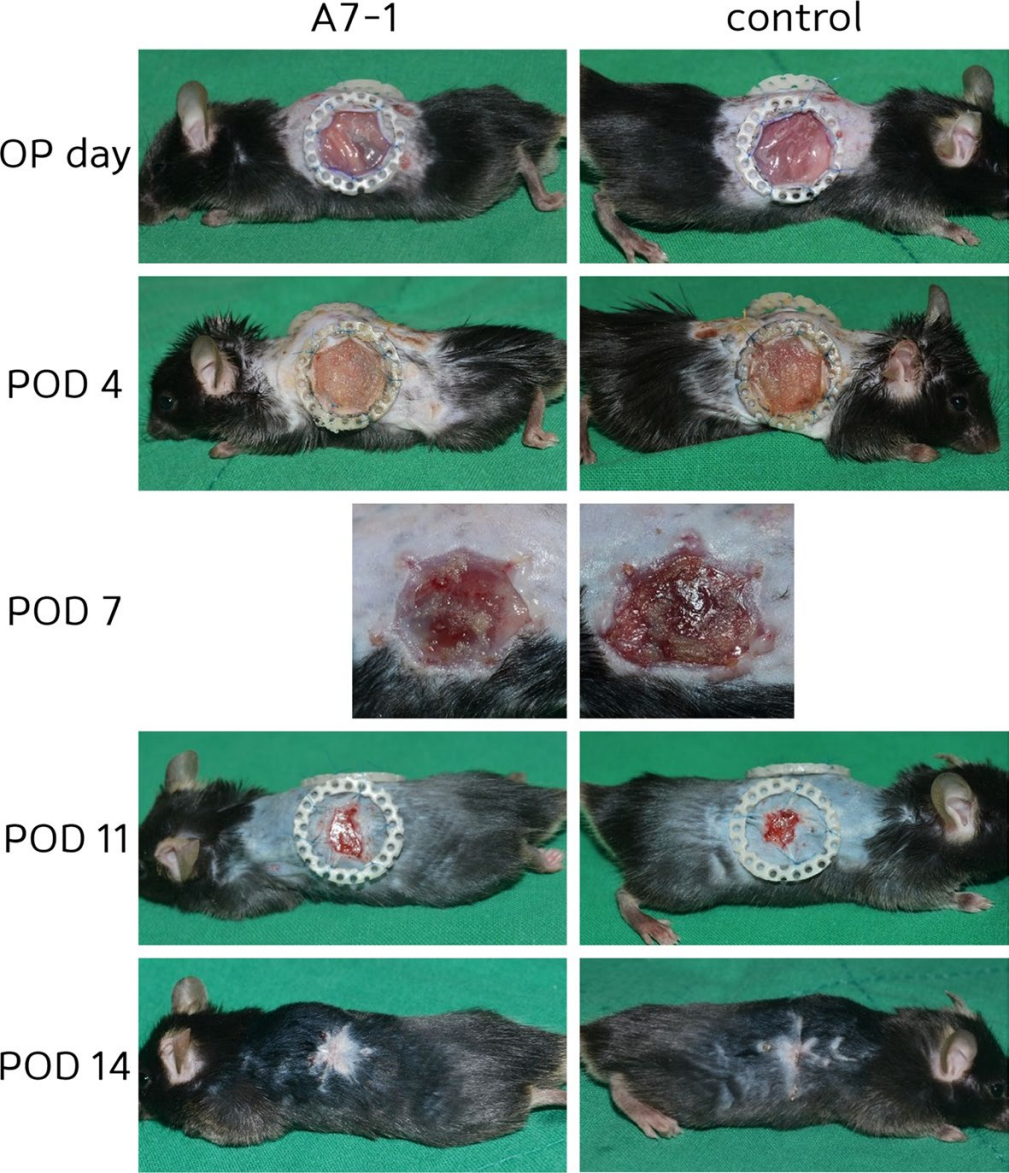
Wound healing-restricted contracture model: Two 15 mm circular skin excisions were made on the backs of mice. Digital photos were taken on days 4 (n = 5), 7 (n = 5), 11 (n = 5), and 14 (n = 5) (total n = 20)

**Fig. 2:**
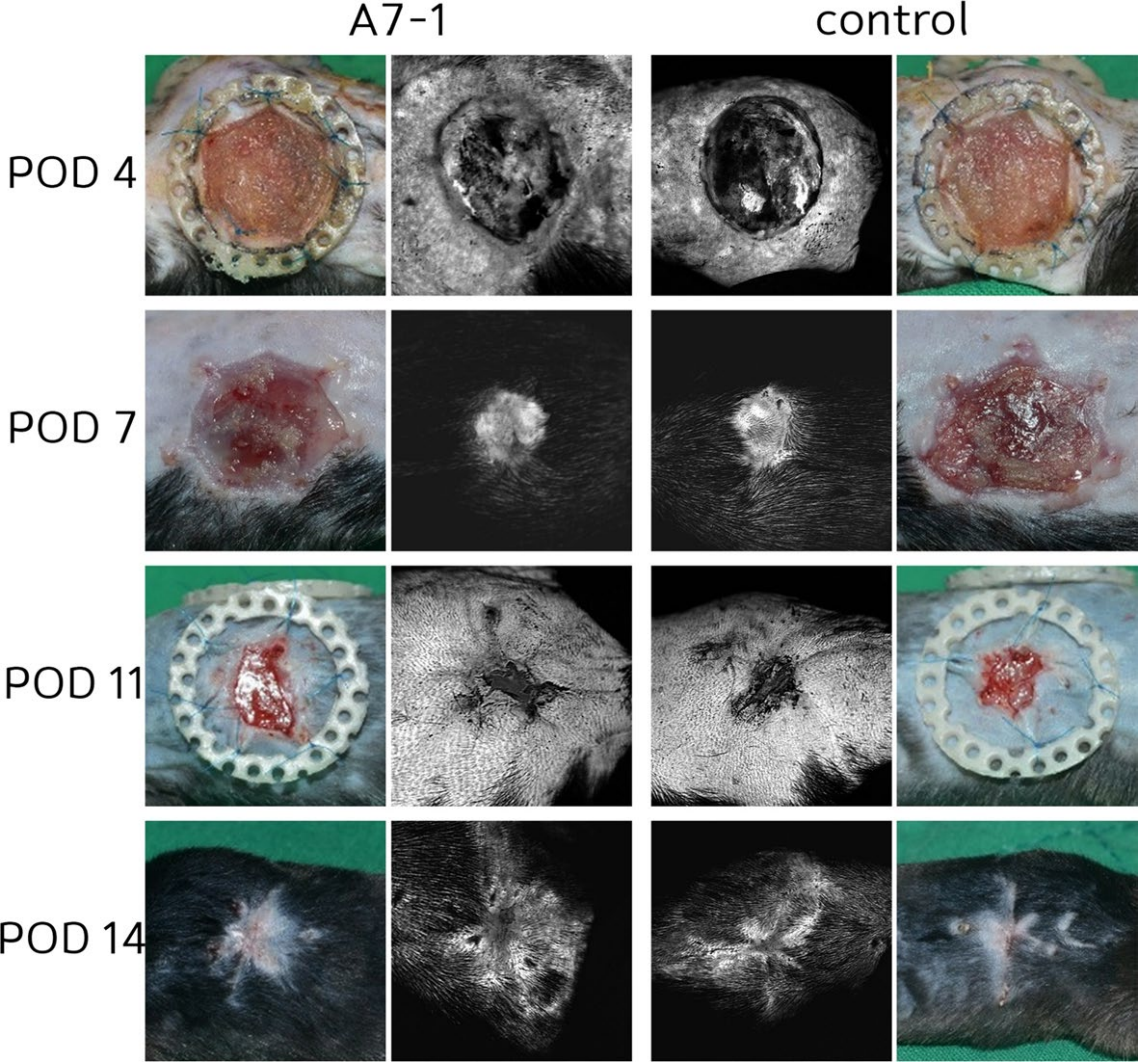
Angiogenesis of wound healing: Angiogenesis was faster in fluorescein angiography examination in wound healing (11%)

In the wound model, the A7-1 group showed a mean wound area of 169.3 mm^2^ on the day of operation and 137.3, 85.2, 40.5, and 1.2 mm^2^ on PODs 4, 7, 11, and 14, respectively. The control group showed a mean wound area of 168.8 on the day of operation and values of 145.3, 111.9, 52.2, and 1.6 mm^2^ on PODs 4, 7, 11, and 14, respectively (Figs. 1, 2).

**Fig. 3:**
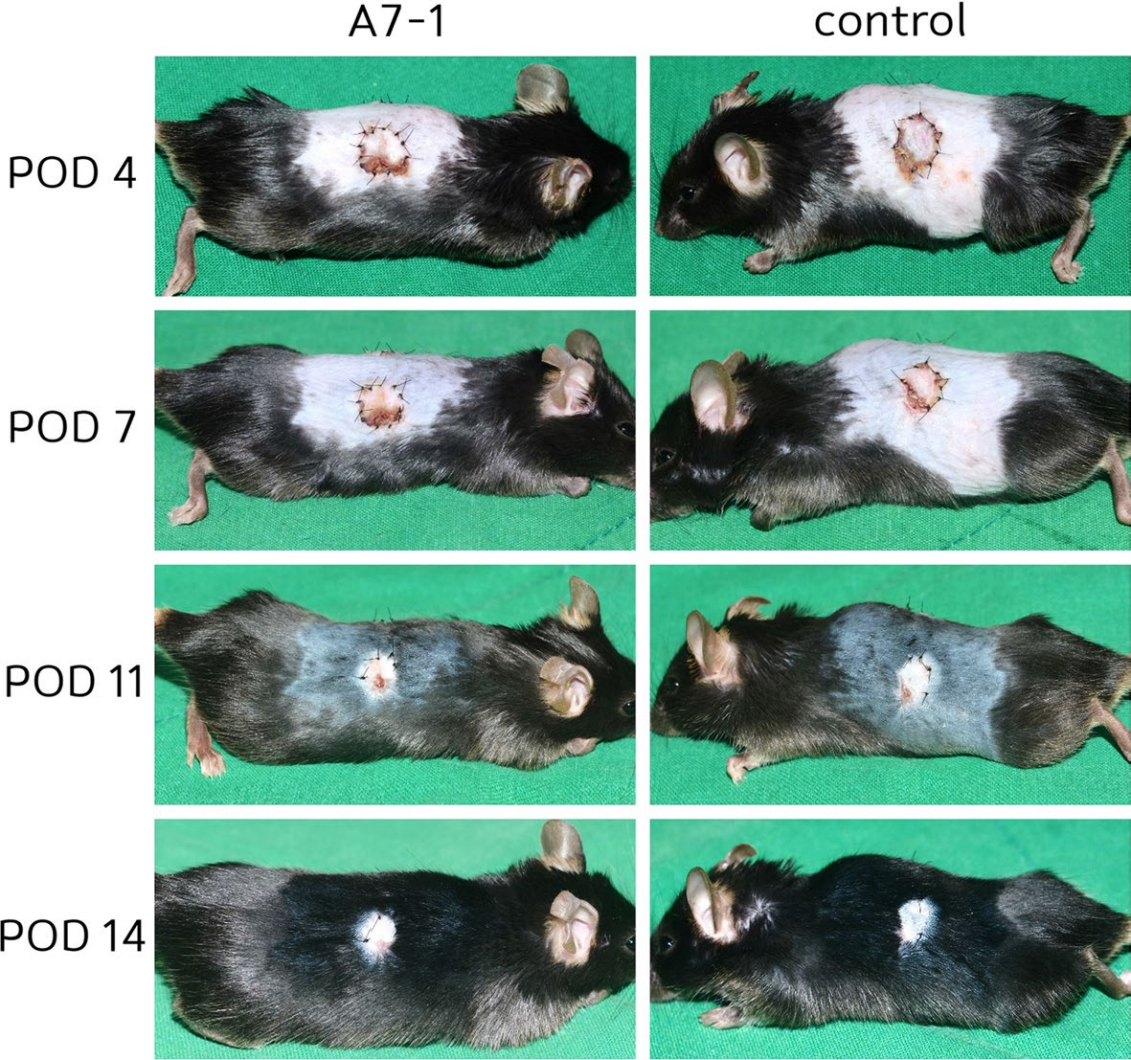
Full-thickness skin graft model: Two circular excisions, exposed to A7-1 and normal saline treatments, were sutured. Digital photos were taken on PODs 4, 7, 11, and 14 (n = 20)

**Fig. 4:**
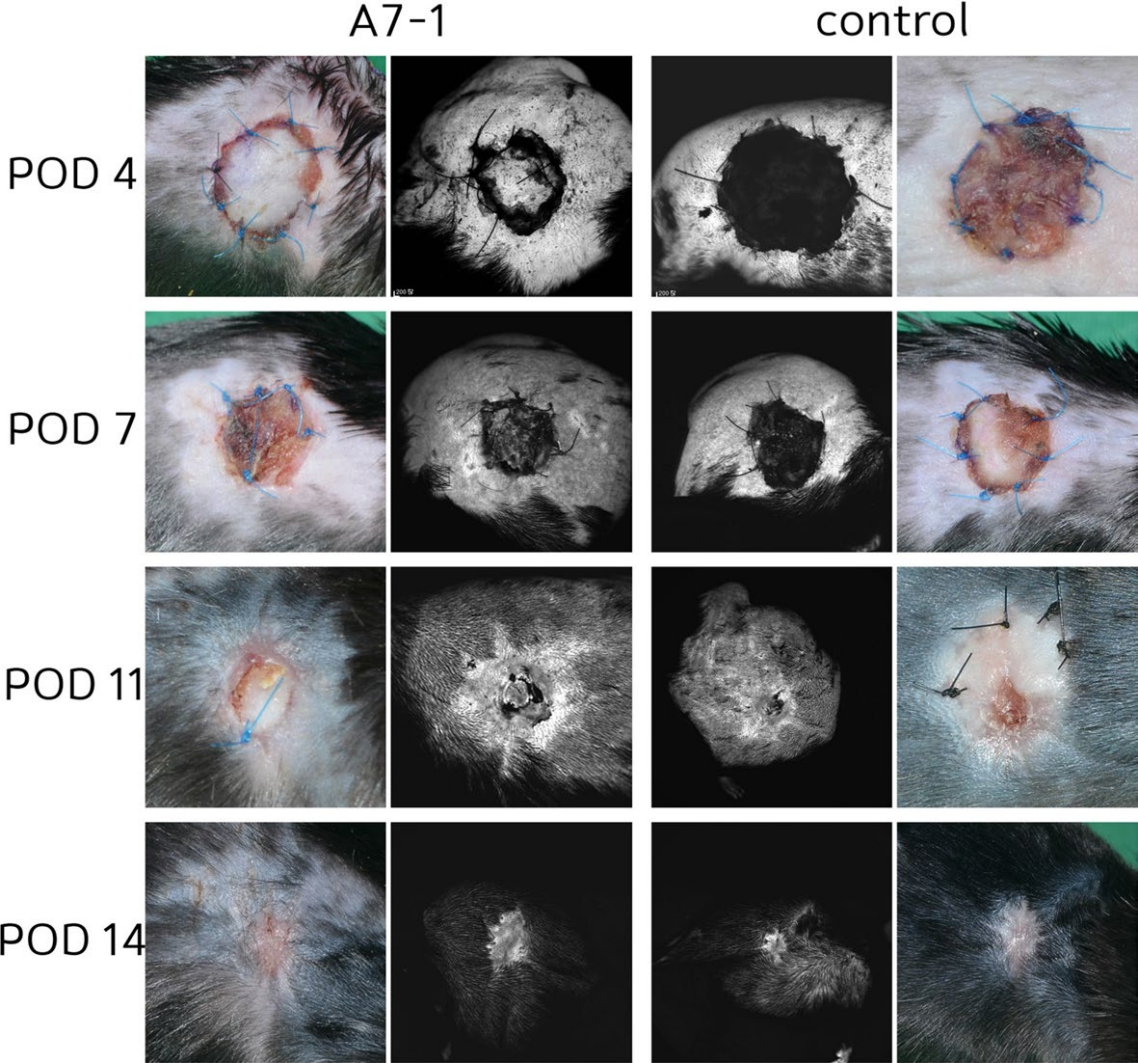
Angiogenesis of skin graft: Angiogenesis was faster in fluorescein angiography examination in skin graft (15%)

In the FTSG model, the area where the graft did not take and cluster area were added together. The A7-1 group showed a mean value of 96.8 mm^2^ on the day of operation and 76.4, 50.2, 22.5, and 3.1 mm^2^ on PODs 4, 7, 11, and 14, respectively. The control group showed a mean value of 96.7 mm^2^ on the day of operation and 80.2, 54.1, 31.8, and 3.3 mm^2^ on POD 4, 7, 11, and 14, respectively (Figs. 3, 4).

**Fig. 5:**
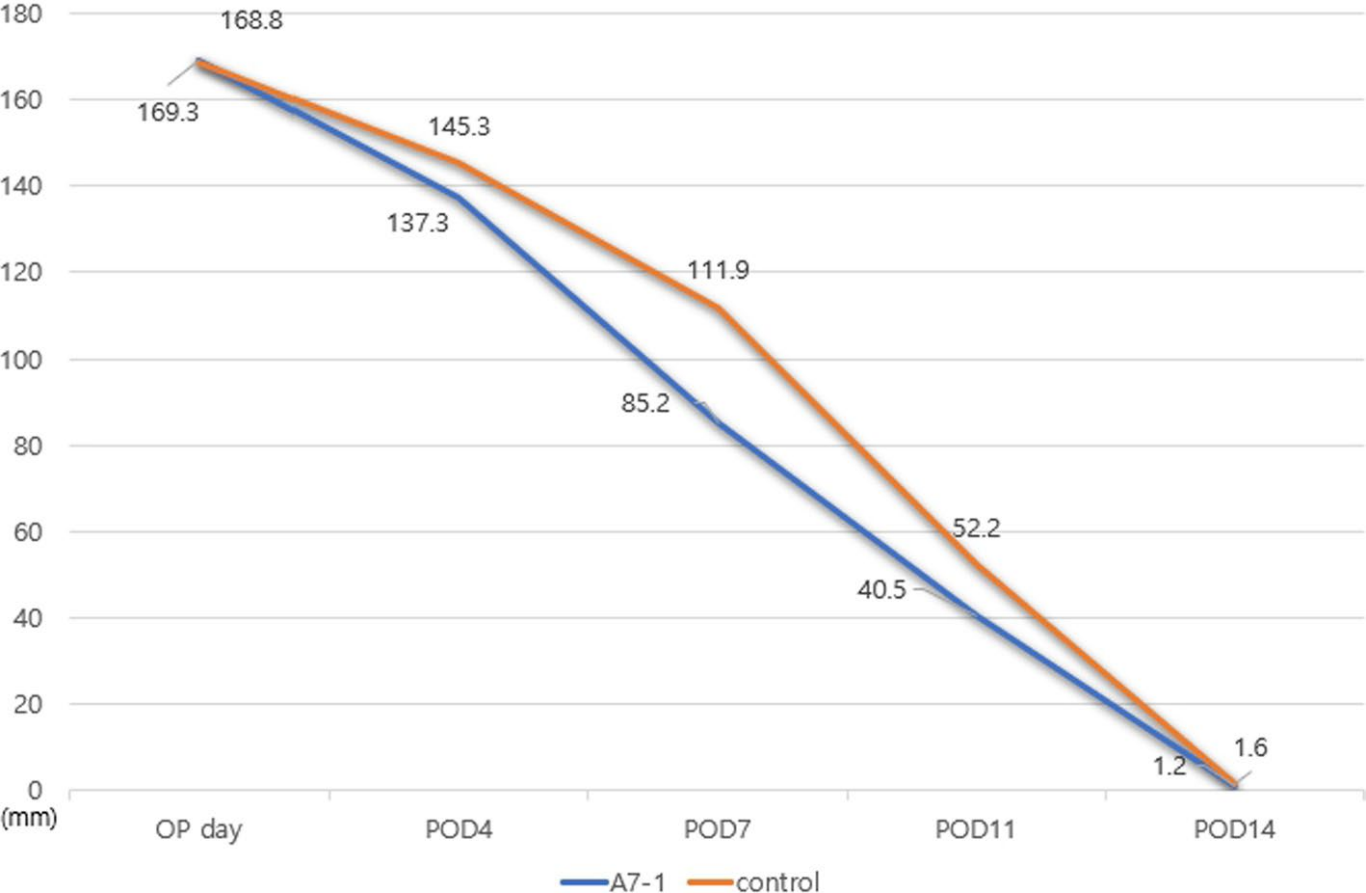
Changes in wound size for healing-restricted contracture model: A7-1 treatment group showed faster wound healing (16.5%)

**Fig. 6:**
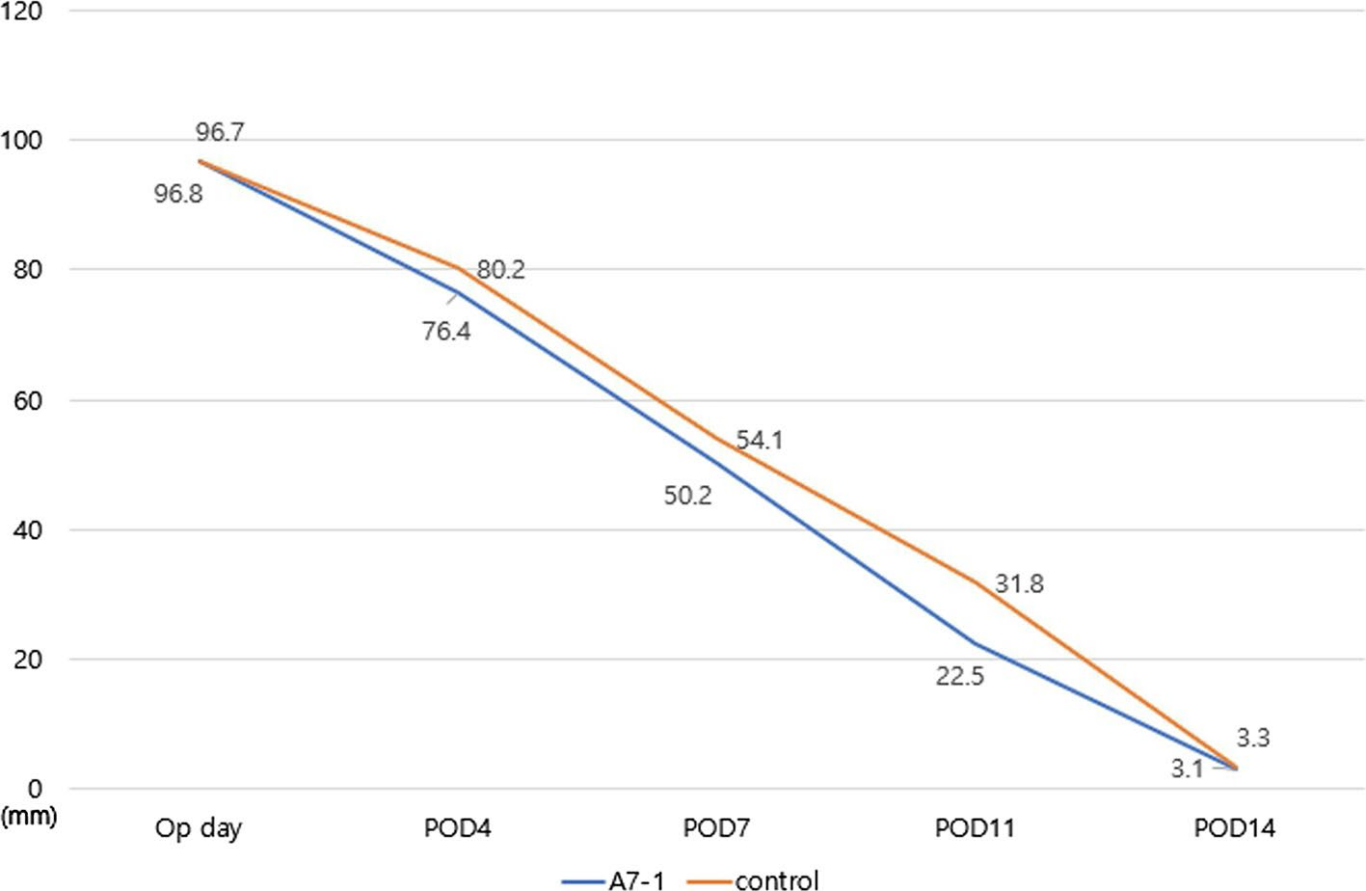
Changes in wound size in full-thickness skin graft model: A7-1 treatment group showed faster skin graft take (17.3%)

To compare the effects of A7-1 with those of the control group, the wound healing rate was defined as [control group]—[A7-1 group]/ [control group] on each day. In the wound model the healing rates were 5.6%, 24.0%, 23.1%, and 9.0% on PODs 4, 7, 11, and 14, respectively, giving a mean value of 16.5%. In the FTSG graft model, the rates were 4.7%, 7.2%, 29.2%, and 6.0% on PODs 4, 7, 11, and 14, respectively, giving a mean value of 17.3% (Figs. 5, 6). In all four groups, there were no infections or other complications.

In the A7-1 experimental and control groups, the wound size of the Wound Healing model and the graft size of the FTSG model were statistically analyzed on the day of surgery and on PODs 4, 7, 11, and 14. The analysis included the calculation of Average, Standard Deviation, Standard Error, and Confidence Interval for each time point (Tables 1, 2).

**Table 1:** Statistical analysis of wound healing model

Wound healing model					
A7-1	Operation day	POD4	POD7	POD11	POD14
Average	169.305	137.365	85.2	40	1.2
SD	3.620582	7.338533	2.034988	3.699428	0.151186
SE	0.849101	1.721036	0.559365	1.280625	0.08
CI	0.050767	0.102898	0.032948	0.073358	0.00424
Control	Operation day	POD4	POD7	POD11	POD14
fx (average)	168.225	145.42	111.9467	52.62	1.6
SD	3.900659	8.020898	7.325858	4.308303	0.232993
SE	0.914354	1.881058	2.013684	1.491898	0.123288
CI	0.109387	0.224933	0.205441	0.120819	0.006534

*SD* Standard Deviation, *SE* Standard Error, *CI* Confidential Interval

**Table 2:** Statistical analysis of FTSG model

FTSG Model					
A7-1	operation day	POD4	POD7	POD11	POD14
Average	96.75	76.395	50.20667	22.47	3.14
SD	6.123439	4.459425	1.82372	2.439693	0.205913
SE	1.369242	0.997158	0.470883	0.771499	0.092087
CI	0.085861	0.062529	0.025572	0.034209	0.002887
Control	Operation day	POD4	POD7	POD11	POD14
Average	96.74	80.155	54.06667	31.83	3.34
SD	5.647247	4.051355	2.696088	3.541765	0.32619
SE	1.262763	0.90591	0.696127	1.120004	0.145877
CI	0.079184	0.056807	0.037804	0.049661	0.004574

*SD* Standard Deviation, *SE* Standard Error, *CI* Confidential Interval

The results of statistical analysis (paired t-test) between the A7-1 group and the control group for mean wound area were significant at POD7 and 11, and in the model for FTSG, significance was found at POD4, 7, and 11 (p < 0.01) (Table 3).

**Table 3:** Statistical analysis(paired t-test) between A7-1 and control group

P-value	OP Day	POD4	POD7	POD11	POD14
Wound healing Model	0.5230	0.1313	0.0025	0.0001	0.4829
FTSG model	0.9968	0.0007	0.0074	0.0002	0.2155

### Confocal laser scanning microscope

**Fig. 7:**
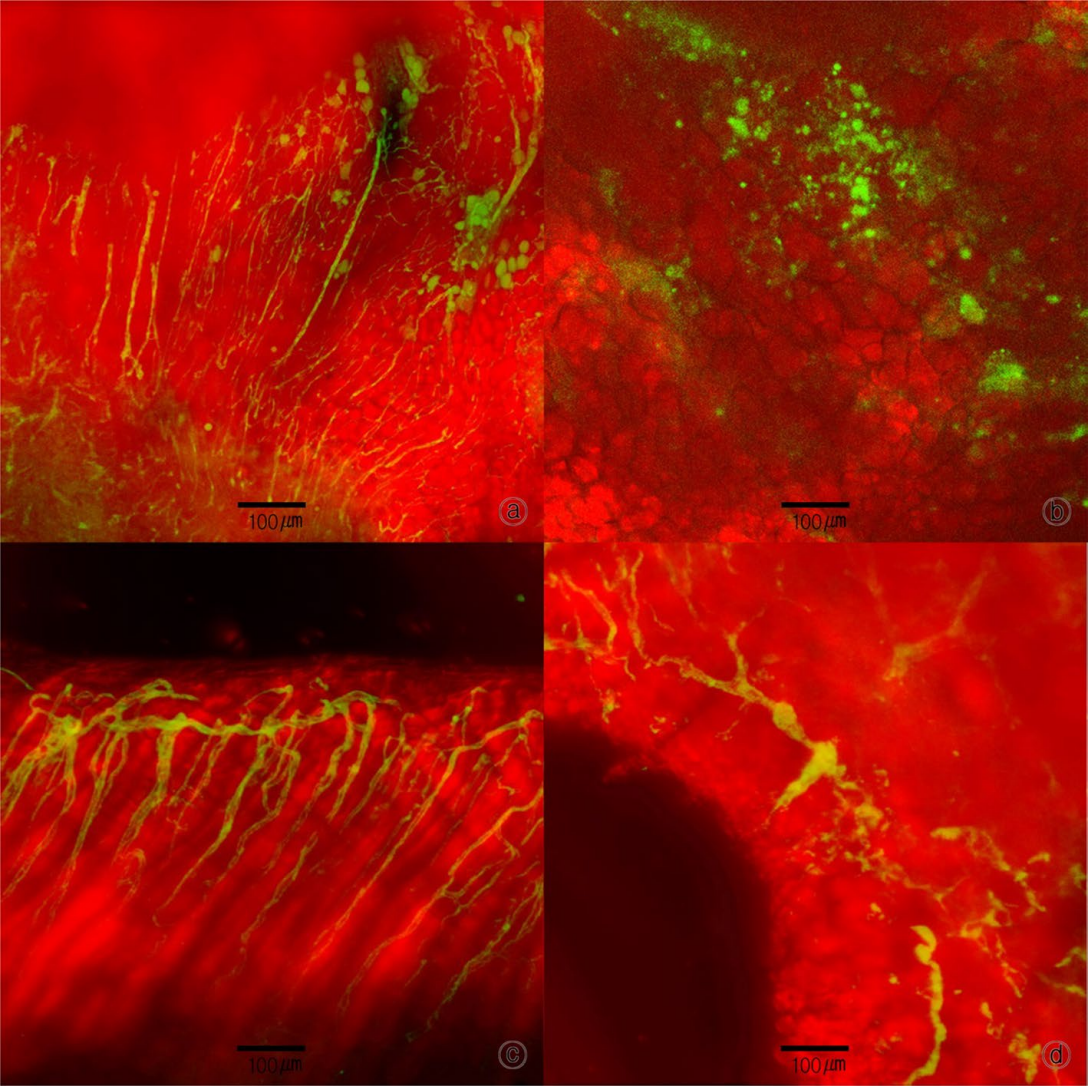
Confocal laser scanning microscopy (skin graft): (a) Experimental group, POD 7 (× 100), (b) control group, POD 7 (× 100), (c) experimental group, POD 14 (× 100), (d) control group, POD 14 (× 100); the rate of angiogenesis was faster and more even in the experimental group than in the control group

On the day of operation and PODs 4, 7, 11, and 14, 300 µL of conjugation to fluorescein isothiocyanate-dextran was injected into the mouse tail vein. After 5 min, the samples were evaluated via confocal microscopy. In both the wound model and FTSG model, the experimental group showed a faster rate of vascular growth, with higher vascularization than the control group. However, because of inconsistencies in the location of image acquisition, the ability to quantitatively analyze the data was limited, although the shape and conditions of angiogenesis could be observed (Fig. 7).

### Laser-induced fluorescein fluoroscopy

The angiogenesis rate was defined as [A7-1 group]−[control group]/ [A7-1 group]. After examining each wound by fluoroscopy, Image J software was used to calculate the mean brightness value. In the wound model, the values were 7.7%, 10.1%, 12.2%, and 15.1% on PODs 4, 7, 11, and 14, respectively, giving a mean value of 11.3%. In the FTSG model, the values were 9.0%, 14.3%, 17.5%, and 20.2% on PODs 4, 7, 11, and 14, respectively, giving a mean value of 15.2%.

### Histological examination

In histological examination, optical microscopy evaluation of reepithelization revealed that in both the wound model and FTSG model, both the A7-1 and control groups showed reepithelization beginning between PODs 4 and 7, which increased rapidly by POD 11. Nearly complete reepithelization was achieved by POD 14.

Infiltration by inflammatory cells, such as polymorphonuclear cells and lymphocytes, in the A7-1 group in the wound model increased between PODs 7 and 11. In contrast, the increase in the control group was not as high as that in the A7-1 group but showed some infiltration of inflammatory cells up to POD 11, which decreased thereafter.

With respect to vascular proliferation, in both the wound model and FTSG model, the A7-1 group showed active angiogenesis between PODs 4 and 7, whereas the control group showed increased angiogenesis between PODs 7 and 11.

**Fig. 8:**
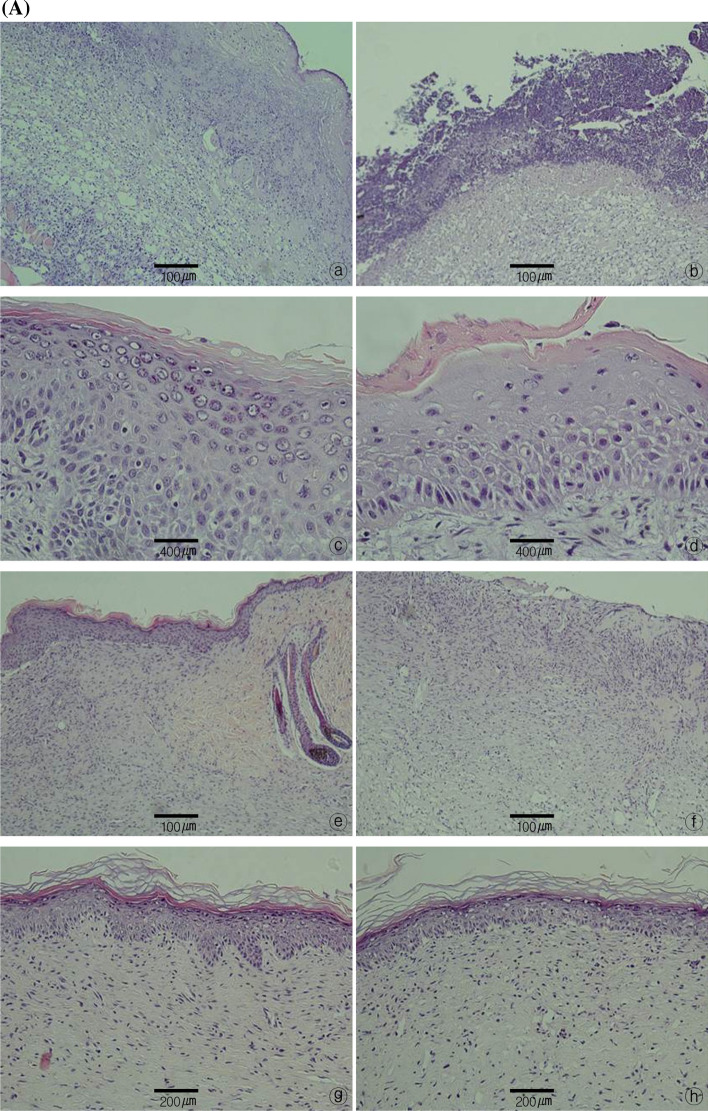

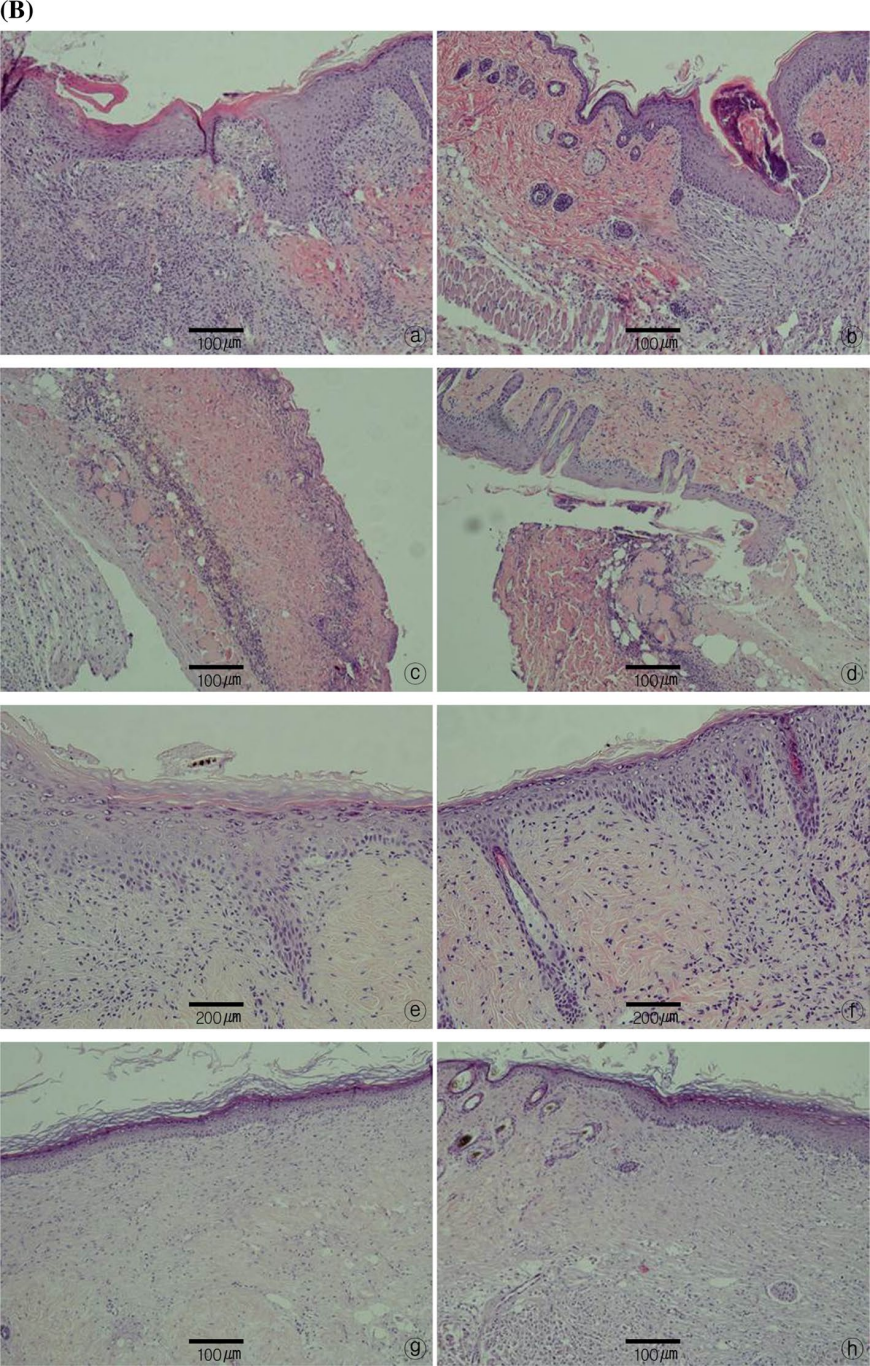
**A** Histological examination of tissue stained by hematoxylin–eosin. Wound healing treatment experimental (a, × 100), control (b, × 100), after 4 days of operation. Experimental (c, × 400), control (d, × 400) after 7 days of operation. Experimental (e, × 100), control (f, × 100) after 11 days of operation. Experimental (g, × 200), control (h, × 200) after 14 days of operation. **B** Histological examination of tissue stained by hematoxylin–eosin. skin graft experimental (a, × 100), control (b, × 100), after 4 days of operation. Experimental (c, × 100), control (d, × 100) after 7 days of operation. Experimental (e, × 200), control (f, × 200) after 11 days of operation. Experimental (g, × 100), control (h, × 100) after 14 days of operation

For collagen fiber formation, the A7-1 group showed weak formation up to POD 7, whereas the control group showed relatively abundant formation of collagen fibers in all wounds by POD 11. By POD 14, all wounds in the A7-1 group showed densely packed and well-aligned collagen fibers and the amount of collagen fibers had increased (Fig. 8A, B).

### Expression of VEGF protein (western blotting)

**Fig. 9:**
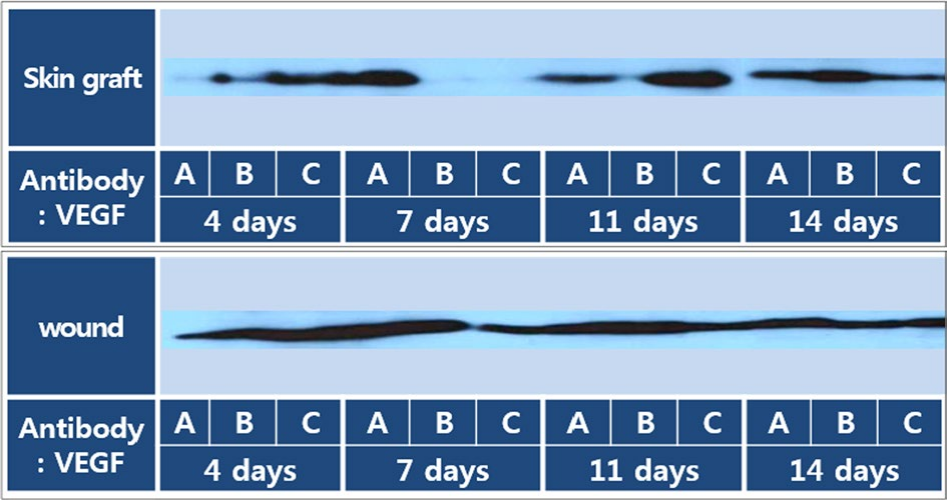
Western blot: (**a**; normal skin, **b**; control, **c**; experimental). Neovascularization (angiogenesis) is the most active and vascular endothelial growth factor (VEGF) and is initially highly expressed in the experimental group

Particularly high levels of VEGF protein expression in the wound model and FTSG model were observed on PODs 4 and 11 (Fig. 9).

## Discussion

The wound healing process can be divided into three main phases: the inflammatory, proliferative, and remodeling phases. It also involves a series of complex processes such as hemostasis, inflammation, angiogenesis, fibrosis, wound contraction, and reepithelization. Various cells are involved in the wound healing process, which is regulated by various cytokines and growth factors [10–12]. When a wound occurs, different types of inflammatory cells, including neutrophils, infiltrate into the wound site to trigger an inflammatory response. During this process, inflammatory cells such as phagocytes produce free radicals that cause oxidative stress in the wound site, resulting in lipid peroxidation, DNA damage, and enzyme inactivation, leading to delayed wound healing [13–15]. The remodeling phase involves complex processes such as the simultaneous occurrence of synthesis and decomposition of cell–cell and cell-extracellular matrix interactions. The extracellular matrix plays various important roles in different phases of wound healing, including the facilitation of phagocytosis and migration of fibroblasts, endothelial, and epithelial cells. Collagen and fibronectin in the extracellular matrix also act as chemotactic factors in fibroblasts [16]. In the wound healing process, the recovery of wound tension is achieved by increasing collagen. Wound tension is directly correlated with the amount of collagen affecting scar formation and intra- and intermolecular bonding of collagen. Adequate recovery of wound tension results in functionally and esthetically ideal wound healing. Wound healing occurred through normal phase experiments conducted by Nasir et al. [17] and Gust et al. [18].

Creating a full-thickness wound on the back of a mouse can lead to natural healing due to contracture and it is difficult to determine how much of that healing can be attributed to contracture. Therefore, a donut-shaped silicon plate (0.5 mm thick) was placed around the wound site and fixed by suturing to prevent contracture [19–22].

When changes in the size of the wound area in both the wound model and FTSG model were observed, the experimental groups showed a pattern of slightly faster reduction in wound area over time, which indicated that A7-1 is effective in facilitating wound healing. Moreover, since A7-1 protein was applied additionally to the wound model in 24-h cycles, it was found to be slightly more effective [22, 23].

Particularly, western blot analysis of tissues from the control and experimental groups showed that expression levels of VEGF protein, a marker involved in angiogenesis, were slightly higher in the experimental group than in the control group [24, 25].

Confocal laser-scanning microscopy is a very useful method for observing angiogenesis in animals. Recently, fluorescent proteins have been used to detect capillaries, arterioles, and venules. In this study, confocal laser-scanning microscopy was used for imaging of the vessels. The results showed differences in the rate of angiogenesis and the formation of new vessels. Although the experimental group showed a faster angiogenesis rate than the control group, the results could not be quantified because of differences in the wound location. Quantitative analysis will be performed in future studies [26–28].

Wound healing involves a series of complex processes such as cell proliferation, migration, and activation, among which the attachment of key cells to the wound site is considered crucial. Therefore, the authors aimed to identify molecules that assist in the attachment of these important cells. As previously described, authors discovered a synthetic peptide called A7-1. The new synthetic peptide, A7-1, demonstrated excellent results in promoting adhesion and remodeling in various cell lines, including C2C12, NIH3T3, MC3T3-E1, HaKaT, ST-2, as well as several primary culture cells. Moreover, using HUVEC cells for angiogenesis and vascularization experiments, A7-1 exhibited results comparable to significant concentrations of VEGF (data not shown). Considering the results from in vivo fluorescein angiogenesis, confocal laser scanning microscope, and biopsy, it can be inferred that A7-1 promotes early attachment of important wound cells, remodeling, and angiogenesis, thus facilitating wound healing. And A7-1 facilitates healing by triggering the proliferation and migration of skin cells within and near the wound by various growth factors and cytokines and by forming a fibrin layer on the wound. Moreover, it promotes active wound healing possibly by improving blood flow through the activation of angiogenesis [29–31].

This study had the following limitations. First, biopsy, fluoroscopy, and western blotting were performed on 5 animals each on PODs 4, 7, 11, and 14. Thus, studies including larger sample sizes are necessary. Second, C57BL/6 mice are readily available and inexpensive, making them easy to care for, rear, and use in experiments. They are therefore valuable as animal models as compared to other animals. Although a method for preventing wound contracture was used, additional studies of larger animals are needed to determine the effects of A7-1 [32, 33]. Clinical trials are also needed to determine the effects of the novel protein A7-1 on wound healing. Third, the novel protein A7-1 has several limitations, such as problems associated with adhesion, fixation, and its limited storage period. To increase its convenience, the absorption of exudates and maintenance in a wet environment must be improved [34, 35].

## Conclusion

The A7-1 treatment group was significantly faster than the control group in wound (17.3%) and skin graft (16.5%) healing. The angiogenesis was significantly faster in fluorescein angiography examination in wound healing (11%) and skin graft (15%) than that of the control group. This study used a wound healing–restricted contracture model and an FTSG model of C57BL/6 mice with wounds created on the back for comparison of the experimental group (A7-1 protein applied) and control group (normal saline applied). The A7-1 group showed faster initial wound healing rates and higher angiogenesis in the early stage. It is expected that the A7-1 protein forms a fibrin layer on the wound and maintains various growth factors and cytokines, which facilitates wound healing. The findings in this study can be used as baseline data for other studies on the wound healing effect of the novel protein A7-1.

## Methods

### Animals

Forty ten-week-old male C57BL/6 mice, with body weights ranging from 25 to 30 g, were utilized in this study. These mice were housed under consistent and controlled laboratory conditions and provided with standard mouse feed and access to water. An acclimation period of one week was observed prior to the initiation of any experiments to ensure that the mice adapted to their environment. Throughout the entire experimental duration, the mice were maintained under conditions of constant temperature and relative humidity. Twenty animals were assigned to each of the two experimental groups: the wound healing model (restricted contracture) group and the full-thickness skin graft model group. Biopsies were performed on specific days following the surgical procedures, namely, on days 4, 7, 11, and 14 after the operation, designated as post-operative day (POD) 4, 7, 11, and 14, respectively. All procedures involving animals in this study were conducted in strict accordance with ethical guidelines and were approved by the Ethics Committee of the Daegu-Gyeongbuk Medical Innovation Foundation. These procedures adhered to the principles outlined in the Declaration of Helsinki, ensuring the ethical treatment and care of the animals involved.

### Wound model-restricted contracture

*Anesthesia and preparation* The procedure began with the administration of inhaled anesthesia using isoflurane obtained from Hana Pharm, Seoul, Korea. Once the mice were appropriately anesthetized, the dorsal area of each animal was carefully shaved to ensure a clean surgical site. Subsequently, the shaved area was sterilized using a 7.5% povidone-iodine solution and alcohol to minimize the risk of infection.

*Creating Circular Wounds* A circular marking with a diameter of 15 mm was made on the dorsal area of the mice using a stamp. Two full-thickness skin defect models were generated within this marked circular area using a #15 surgical blade. The two wounds, each measuring 15 mm in size, were precisely created by removing the entire skin layer down to the level of the panniculus carnosus, which is approximately 0.2 cm thick. This ensured uniformity in the wound creation process. To prevent contamination from surrounding areas, sterile drapes were meticulously applied to cover regions other than the dorsal area.

**Fig. 10:**
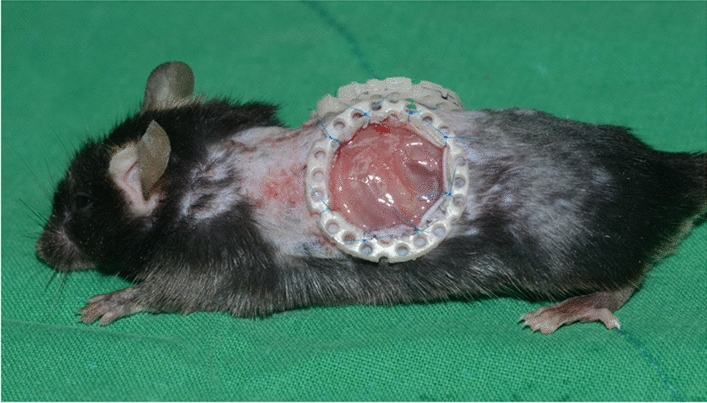
Production of a wound healing-restricted contracture model: A stamp, 15 mm in diameter, was used to create two full-thickness defects on the dorsal area. A donut-shaped silicon plate (0.5 mm thick) was placed near the wound site and fixed by suturing to prevent the wound from contracting

*Preventing Contracture* To prevent wound healing through contracture, a donut-shaped silicone plate, 0.5 mm thick, was employed. This silicone plate featured a central hole with a diameter of 18 mm. It was securely fixed in place using Ethilon #5 sutures [9]; (Fig. 10). This step was crucial in maintaining the wound’s intended characteristics.

*Experimental and Control Groups* Among the two defect sites created, one was randomly designated as the experimental group, while the other served as the control group. To distinguish between the two, the ear on the same side as the experimental group was marked accordingly.

*Application of A7-1 Protein* In the experimental group, the novel A7-1 protein (100 µM, 2 cc) was applied to the wound site at 24-h intervals after the surgical operation. This application was performed to assess the protein’s impact on wound healing. In contrast, the control group’s wound received applications of normal saline (2 cc) using the same schedule. This distinction allowed for a comparative evaluation of the effects of A7-1 protein on wound healing.

### Production of full-thickness skin graft (FTSG) model

*Anesthesia and preparation* The procedure commenced with the administration of inhaled anesthesia using isoflurane, supplied by Hana Pharm in Korea. Following anesthesia, the dorsal area of each mouse was meticulously shaved to provide a clean surgical site. Subsequently, the shaved area was thoroughly sterilized using a 7.5% povidone-iodine solution and alcohol to minimize the risk of infection.

*Creating full-thickness excision sites* to establish the graft model, a circular mark with a diameter of 15 mm was carefully drawn on the dorsal area of the mice. This marked area served as the site for the creation of two full-thickness skin defect models. Two full-thickness skin defect models were generated within the marked circular area. These excisions ensured that the entire thickness of the skin was removed.

*Experimental and control groups* After the creation of the skin defects, one side was randomly assigned as the experimental group, while the other served as the control group. To distinguish between the two groups, the ear on the same side as the experimental group was marked accordingly.

*Ensuring uniform graft thickness* It was essential to maintain uniformity in the thickness of the skin grafts harvested. To achieve this, an even amount of tension was applied to the donor surface.

*Application of A7-1 protein and suturing* In the experimental group, the novel A7-1 protein (100 µM, 2 cc) was applied to the graft site. Subsequently, the skin that had been removed was sutured back in place using 5–0 Ethilon sutures. This process aimed to assess the impact of A7-1 protein on the graft site. In the control group, normal saline (2 cc) was applied to the graft site using the same method. The skin was then sutured back into place using 5-0 Ethilon sutures [5].

### Wound healing rate

*Image acquisition* After inducing the wounds in both the wound and full-thickness skin graft (FTSG) model, images were captured on specific days. These days included the day of the surgical operation and PODs 4, 7, 11, and 14. To capture high-quality images, a stereoscopic zoom microscope (SMZ 745 T, Nikon, Tokyo, Japan) was employed.

*Image analysis* The acquired images of the wound and graft sites were analyzed using an image analysis program called Image J (NIH, Bethesda, MD, USA). This software facilitated accurate measurement of the wound area and graft site.

*Biopsy* Tissue biopsies were conducted on select animals at different time points. Specifically, biopsies were performed on 5 animals from a total of 20 animals on POD 4, 15 animals on POD 7, 10 animals on POD 11, and 5 animals on POD 14. This biopsy strategy allowed for the collection of tissue samples at various stages of the healing process, providing valuable insights [4].

*Consistency in image acquisition* To ensure the reliability of the acquired images and measurements and eliminate potential variations in image quality, it was imperative that consistency was maintained. Therefore, all images were captured by the same researcher in the same location under identical lighting conditions.

*Measurement parameters* In the wound model, measurements focused on assessing the raw surface area of the wound site where healing had not occurred, reflecting the absence of re-epithelialization. In contrast, the FTSG model involved measuring the area where the graft had not healed, indicating a lack of graft acceptance, as well as assessing cluster areas.

*Complete treatment point* The point at which a wound or graft was considered completely treated was defined as the stage of reepithelialization where no further dressing application was required. This marked the successful conclusion of the healing process.

### Laser-induced fluorescein fluoroscopy

To evaluate angiogenesis, laser-induced fluorescein fluoroscopy was employed, following this protocol:

*Anesthesia* On PODs 4, 7, 11, and 14, anesthesia was induced in 5 mice for each time point. This was achieved through the intraperitoneal injection of 0.1 cc of ketamine. Anesthesia ensured that the mice remained immobile during the imaging procedure.

*Fluorescein injection* Following anesthesia, 0.1 mm of a 10% fluorescein solution was injected into the tail vein of each mouse. The fluorescein solution used in this procedure consisted of 500 mg of 10% fluorescein sodium, obtained from Unimed Pharma, Inc. in Seoul, Korea.

**Fig. 11:**
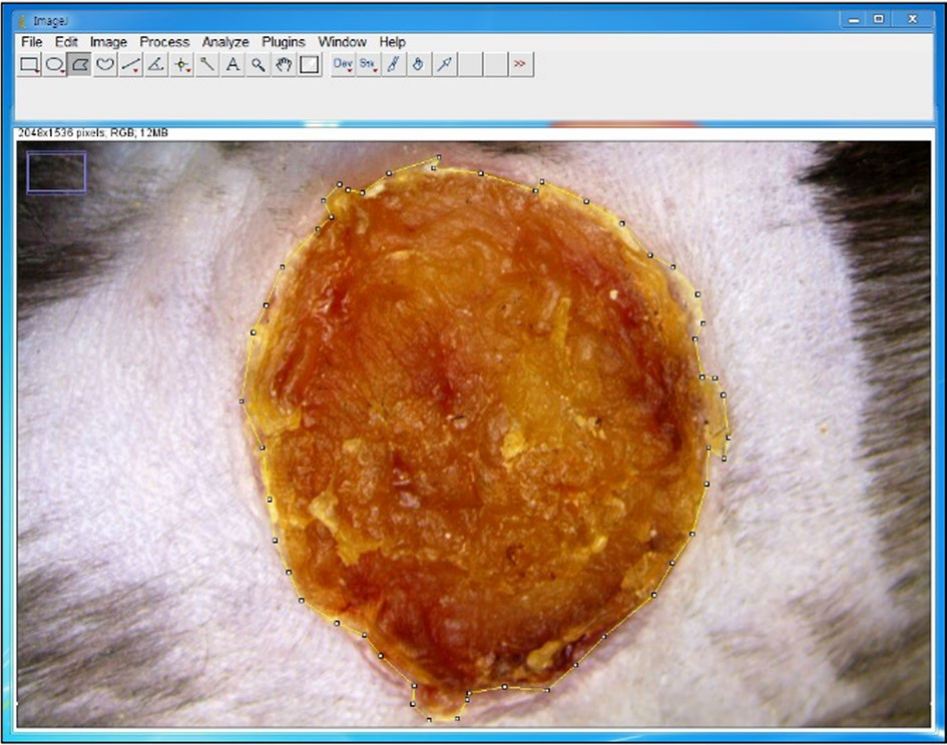
Stereoscopic image of wound for analysis of Angiogenesis using Laser-induced fluorescein fluoroscopy

*Real-Time angiogenesis analysis* One minute after the administration of fluorescein, the degree of angiogenesis was assessed through real-time analysis. This analysis was conducted using the HEIDELBERG Retina Angiogram Digital Angiography System (HRA). The HRA system facilitated the visualization and examination of angiogenesis at the wound site (Fig. 11).

**Fig. 12:**
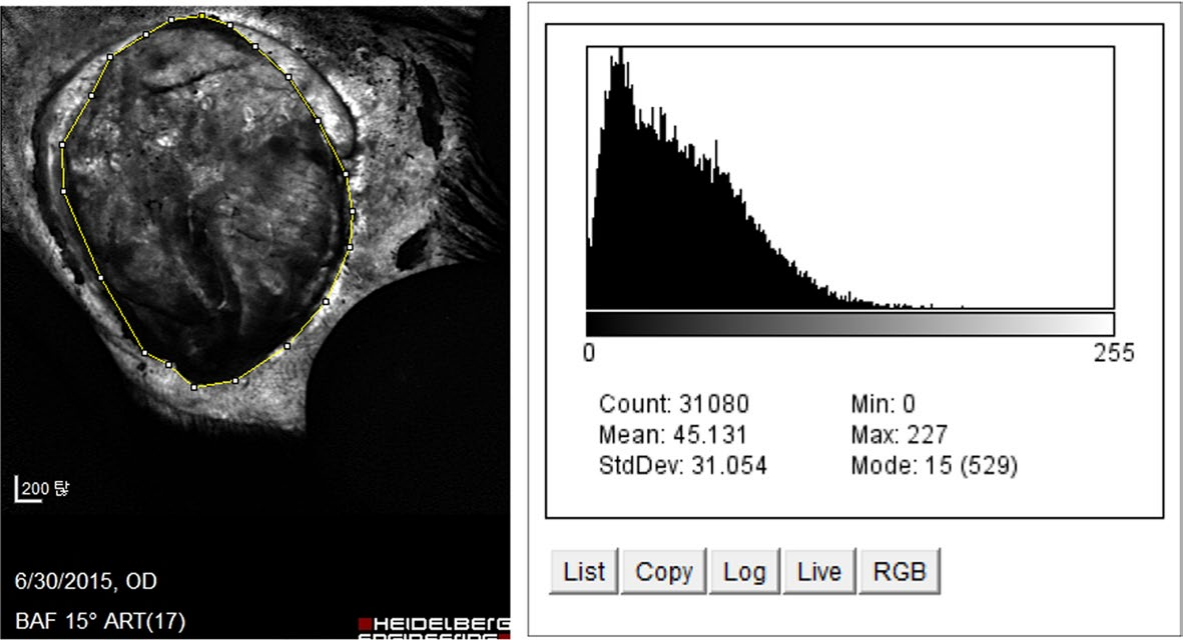
Wound site brightness after fluoroscopy was measured using Image J software

*Image analysis* For a thorough evaluation of the angiogenesis, Image J software was employed. This software enabled the comparison and analysis of the brightness of the images obtained during the angiography process [11]; (Fig. 12). This approach allowed for the quantification and assessment of angiogenesis levels at specific time points in the wound healing process. It provided valuable insights into the development of blood vessels in the wound area, aiding in the understanding of tissue repair and regeneration.

### Confocal laser scanning microscopy

To further assess angiogenesis and observe the appearance and status of angiogenesis, a confocal laser scanning microscope was utilized. The procedure is as follows:

*Post-Fluoroscopy angiogenesis assessment* After the evaluation of angiogenesis through laser-induced fluorescein fluoroscopy on PODs 4, 7, 11, and 14, additional steps were taken to gain more detailed insights into angiogenesis [9].

*Injection of fluorescein isothiocyanate-dextran* On these specified days, 300 µL of a solution conjugated with fluorescein isothiocyanate-dextran was injected into the mouse tail vein. This injection aimed to label blood vessels and visualize their structure and density in the wound area.

Tissue Biopsy: 5 min after the injection of the fluorescein isothiocyanate-dextran solution, a skin biopsy was performed. The biopsy included not only the wound site but also a 4-mm margin of healthy skin surrounding the wound. This comprehensive sampling allowed for the examination of both the wound area and its immediate surroundings.

*Confocal microscopy analysis* The collected tissue samples were immediately analyzed using a confocal laser scanning microscope. This specialized microscope enabled researchers to obtain high-resolution images of the skin tissue, focusing specifically on the appearance and status of angiogenesis.

This confocal microscopy procedure provided detailed visual information about the development and characteristics of blood vessels within the wound and surrounding tissue. It offered a more in-depth perspective on angiogenesis, enhancing the overall understanding of vascular changes during the healing process.

### Histological examination

After observing the blood vessels by confocal laser scanning microscopy, the same animals were sacrificed on PODs 4, 7, 11, and 14 and the skin area that included the skin defect and 4 mm of healthy skin around the wound site was excised up to the panniculus carnosus. The harvested tissue was fixed in formalin, and then the center of the wound site was cut cross-sectionally to prepare a paraffin block, which was sliced into 3-μm slices for hematoxylin and eosin and Masson’s trichrome staining. The degree of reepithelization, proliferation of granulation tissue, necrosis, degree of inflammation, and pattern of angiogenesis were measured by optical microscopy at 40 × and 100 × magnification.

### Vascular endothelial growth factor(VEGF) western blot

*Tissue harvest and storage* Tissue samples were collected from mice that had been sacrificed on PODs 4, 7, 11, and 14. These tissue samples were immediately freeze-stored at −70 °C to preserve their protein content.

*Tissue preparation* To quantitatively analyze VEGF, the frozen tissue samples were removed from storage and added to 0.5 mL of cell lysis buffer. The cell lysis buffer contained the following components: 1% Triton, 1% cholic acid, 50 mM NaCl, and 20 mM Tris–HCl (pH 7.4). Additionally, protease inhibitors, including 1 µg leupeptin, 1 µg pepstatin A, 1 µg aprotinin, and 1 mM PMSF, were added to the buffer. The entire process was conducted at 4 °C to maintain protein stability.

*Tissue homogenization* a tissue homogenizer (Ultra-Turrax T25, IKA-Labor Technique, IKA Process, Wilmington, NC, USA) was used to mechanically disrupt and homogenize the tissue. This step was performed three times for 1 min each time to ensure thorough tissue decomposition.

*Incubation* After tissue homogenization, the samples were incubated at room temperature for 1 h. This incubation allowed for the proper extraction of proteins from the cells and tissue components.

*Centrifugation* Following incubation, the solution was subjected to centrifugation at 15,000 rpm and 15 °C for 30 min. This centrifugation step resulted in the separation of the supernatant, which contained the extracted proteins, from the cellular and tissue debris.

*Reference standard* Bovine serum albumin was used as the reference standard sample for quantification.

*Polyacrylamide Gel Electrophoresis* A portion of the extracted protein supernatant (25 µg) was separated by 10% polyacrylamide gel electrophoresis for 1 h. This separation step allowed the proteins to be separated based on their molecular weights.

*Protein transfer* Following electrophoresis, the proteins were transferred from the gel to a nitrocellulose membrane. This transfer step facilitated subsequent antibody detection.

*Blocking* The nitrocellulose membrane was treated with a blocking buffer for 1 h. The blocking buffer contained 5% nonfat dry milk in a buffer solution composed of 10 mM Tris HCl, 0.15 M NaCl, and 0.1% sodium azide. Blocking helped prevent the non-specific binding of antibodies.

*Primary antibody incubation* The primary mouse antibody for VEGF (sc-7269, Santa Cruz Biotechnology, Dallas, TX, USA) was diluted to a 1:1,000 concentration in the blocking buffer. The membrane was then incubated with the primary antibody for 1 day at 4 °C.

*Secondary antibody incubation* Following incubation with the primary antibody, a secondary antibody bound to horseradish peroxidase was applied to the membrane and allowed to react for 1 h at room temperature.

*Washing* the membrane was washed three times for 10 min each with Tris-buffered saline with Tween 20 (TTBS) to remove unbound antibodies.

*Enhanced chemiluminescence (ECL) Staining* ECL staining was performed to visualize and quantify the proteins of interest. ECL is a chemiluminescent reaction that produces light upon exposure to the target protein-antibody complex.

This comprehensive western blot analysis allowed for the quantitative assessment of VEGF protein levels in the tissue samples, providing valuable data on the expression of this critical growth factor during the wound healing process [9].

### Supplementary Information

Supplementary material 1

Supplementary material 2

Supplementary material 3

## Data Availability

Data sharing not applicable to this article as no datasets were generated or analyzed during the current study.
