# Supplementary figures and images for: Wound healing induced by new synthetic peptide, A7-1, in C57BL/6 mouse model
Source: Biomed Eng Online. 2024 Jul 29;23:75. doi: 10.1186/s12938-024-01247-7 (PMC11285448; doi:10.1186/s12938-024-01247-7)

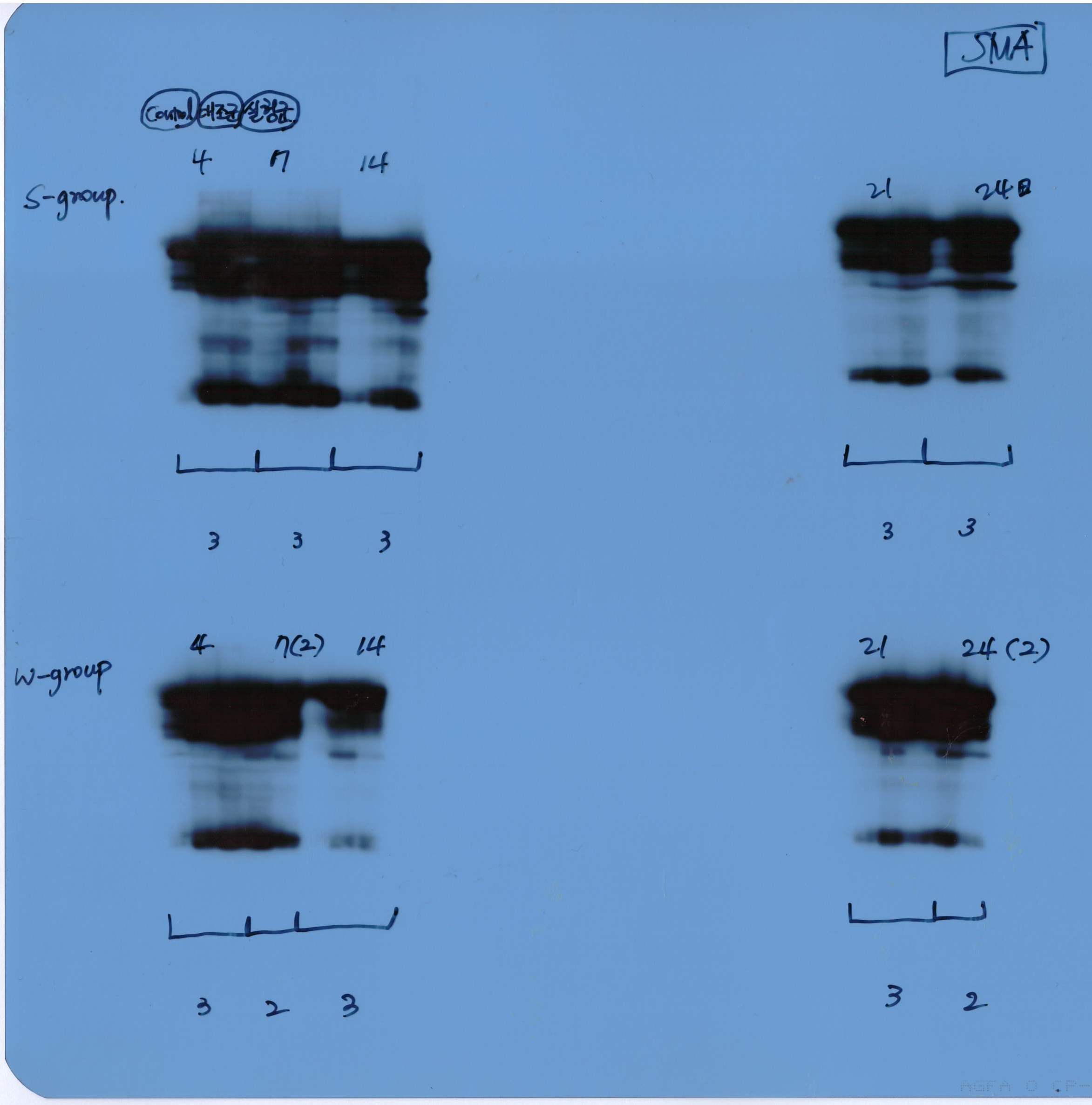

Supplement: Supplementary file 1 — Supplementary material 1 [file 12938_2024_1247_MOESM1_ESM.jpg]

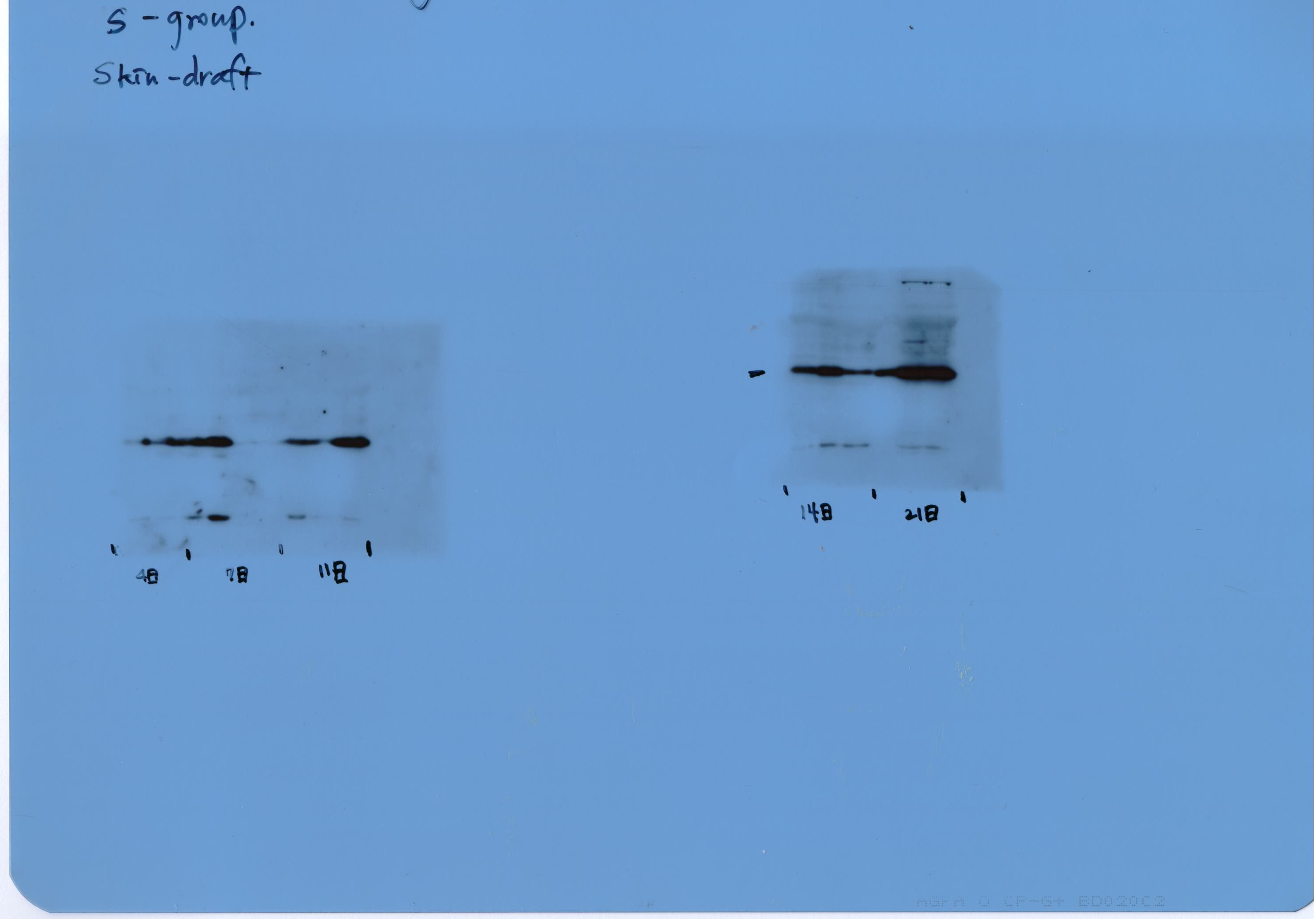

Supplement: Supplementary file 2 — Supplementary material 2 [file 12938_2024_1247_MOESM2_ESM.jpg]

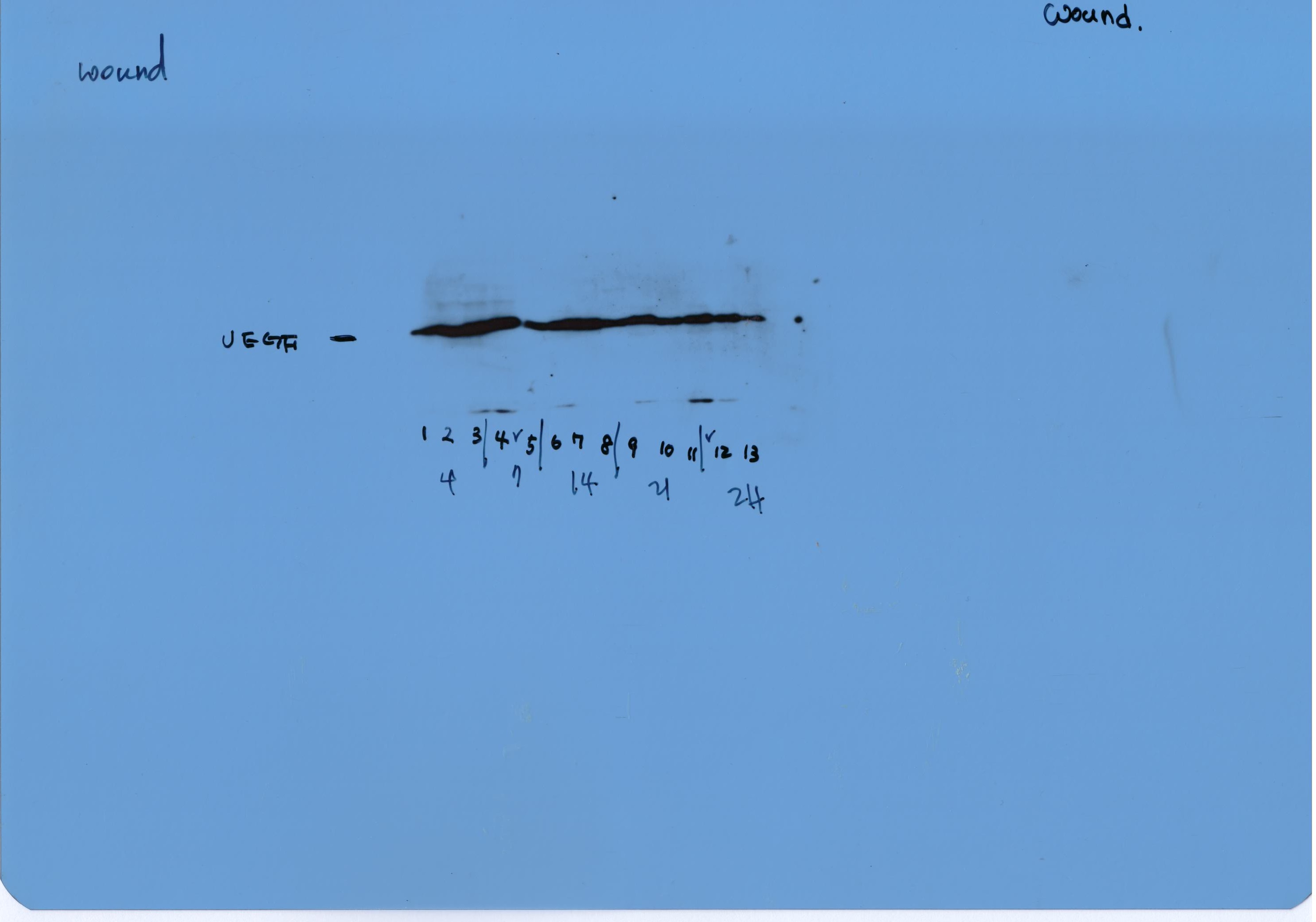

Supplement: Supplementary file 3 — Supplementary material 3 [file 12938_2024_1247_MOESM3_ESM.jpg]
